# Proline Promotes Drought Tolerance in Maize

**DOI:** 10.3390/biology14010041

**Published:** 2025-01-07

**Authors:** Pirzada Khan, Ashraf M. M. Abdelbacki, Mohammed Albaqami, Rahmatullah Jan, Kyung-Min Kim

**Affiliations:** 1 Biotechnology Research Institute, Chinese Academy of Agriculture Sciences, Beijing 100081, China; pirzada_111@yahoo.com; 2 Deanship of Skill Development, King Saud University, P.O. Box 2455, Riyadh 11451, Saudi Arabia; aabdelbacki@ksu.edu.sa; 3 Department of Botany and Microbiology, College of Science, King Saud University, P.O. Box 2455, Riyadh 11451, Saudi Arabia; mmbaqami@ksu.edu.sa; 4 Coastal Agriculture Research Institute, Kyungpook National University, Daegu 41566, Republic of Korea; 5 Department of Applied Biosciences, Kyungpook National University, Daegu 41566, Republic of Korea

**Keywords:** drought stress, proline supplementation, oxidative stress, antioxidant defense, osmoprotection

## Abstract

Drought stress significantly affects maize (*Zea mays* L.) growth by disrupting vital physiological and biochemical processes. This study investigates the potential of proline supplementation to alleviate drought-induced stress in maize plants. The results show that proline supplementation enhanced shoot and root growth under normal conditions and alleviated drought-induced reductions in growth parameters. Under drought stress, proline increased shoot length by 40%, root length by 36%, shoot fresh weight by 97%, root fresh weight by 247%, shoot dry weight by 77%, and root dry weight by 154% compared to the untreated plants. While drought stress induced electrolyte leakage and reduced the relative water content (RWC) and leaf area, proline treatment mitigated these effects by improving membrane stability, water retention, and chlorophyll content. Moreover, proline supplementation reduced hydrogen peroxide (H_2_O_2_) and malondialdehyde (MDA) levels by 38% and 67%, respectively, in the drought-stressed plants compared to the untreated controls. It also enhanced catalase (CAT), peroxidase (POD), and superoxide dismutase (SOD) activities by 14%, 69%, and 144%, respectively, under drought stress, indicating a strengthened antioxidative defense. Proline also increased the protein content and improved N, P, and K retention by 30%, 40%, and 28%, respectively, in the drought-stressed plants, supporting metabolic and osmotic balance. Additionally, proline improved endogenous proline and sugar levels, facilitating osmotic adjustment and providing energy reserves. These findings suggest that proline supplementation effectively enhances maize resilience under drought stress, improving growth, reducing oxidative stress, and enhancing osmoprotection.

## Simple Summary

This research highlights how proline supplementation can help maize plants survive drought, a period of reduced water availability. Proline improved the growth of shoots and roots under normal conditions and helped to sustain this growth under drought conditions. During drought, proline significantly increased the length and weight of both shoots and roots. It also minimized cell damage, maintained water levels, and preserved the chlorophyll content. Additionally, proline reduced the levels of harmful substances like hydrogen peroxide and malondialdehyde while boosting antioxidant enzyme activities in maize plants. It also enhanced the plants’ protein content, nutrient retention, and internal reserves of proline and sugars, supporting osmotic balance. Overall, proline helped maize plants to grow better under drought conditions by alleviating stress, promoting growth, and optimizing water and nutrient management.

## 1. Introduction

Maize (*Zea mays* L.) is a vital crop cultivated worldwide under diverse climatic conditions [1], serving as a staple food for millions, as well as a key resource in animal feed and various industrial applications [2]. However, maize growth is often impeded by several environmental factors, such as salt stress, extreme temperatures (both high and low), toxic metal exposure, UV radiation, and drought stress [3], all of which adversely affect plant growth and productivity depending on the severity of the stress. In response to these stresses, plants have developed a range of physiological and biochemical mechanisms to increase their tolerance and survival.

Among these stresses, water deficit, or drought stress, is a major abiotic factor that severely limits plant growth and yield. Drought stress induces the production of reactive oxygen species (ROS), including hydroxyl radicals (‧OH), superoxide anions (O_2_¯), and hydrogen peroxide (H_2_O_2_). The overaccumulation of ROS leads to oxidative stress, causing significant damage through membrane lipid peroxidation [4], weakening the plant’s defense system, and resulting in protein oxidation, nucleic acid damage, enzyme inhibition, and, ultimately, programmed cell death [5]. Drought stress is particularly detrimental to crops, as it decreases their quality, yield, and stability, especially when experienced during critical stages of growth [6]. In rice and other crops, drought negatively impacts the initiation of flowering, leading to panicle sterility and reduced yields [7]. With the increasing global demand for food, developing drought-tolerant varieties has become crucial. Understanding the biochemical and physiological mechanisms that enable plants to withstand drought stress is essential for selecting and breeding crops with enhanced drought resilience [8]. Under drought stress, plants employ various strategies to cope, including evading drought conditions and enhancing tolerance through specific adaptations. Osmotic adjustment, in particular, is a key physiological process critical for maintaining plant growth under drought stress [9].

Several studies, as summarized by Qasim Ali et al., have reported that plants accumulate various osmoprotectants, such as proline, to enhance their tolerance to both salt and drought stress [8]. Proline functions as a key osmoprotectant, stabilizing protein structures and the photosynthetic apparatus [10]. It also aids in regulating cellular osmotic balance and mitigating the effects of ROS during abiotic stress conditions [11,12,13,14]. Proline scavenges ROS, including H_2_O_2_ and O_2_¯, generated during drought stress, thereby alleviating oxidative stress and protecting the plant from associated damage [15]. Additionally, proline enhances turgor potential, photosynthetic activity, and antioxidant activity, resulting in reduced oxidative damage [16]. Yang et al. [17] and Ihtisham et al. [18] have shown that proline can effectively mitigate the detrimental effects of oxidative stress in plants. Plants that accumulate higher levels of proline demonstrate enhanced stress tolerance [19,20], promoting growth by reducing nutrient imbalances, lowering elemental toxicity, and enhancing photosynthesis [21]. Exogenous proline application during the seedling and vegetative stages of maize has been shown to promote growth under drought conditions [8]. Furthermore, pre-sowing seed soaking with proline has been reported to enhance growth in *Triticum aestivum* under drought stress [22]. Proline supplementation also helps to maintain optimal nutrient (potassium, calcium, phosphorus, and nitrogen) levels in maize, contributing to improved drought tolerance [23]. Potassium is particularly vital for key physiological processes in plants, including stomatal function, transpiration, photosynthesis, photophosphorylation, turgor pressure maintenance, enzyme activation, and photoassimilate transport [24]. Recent studies indicate that applying potassium externally can mitigate the adverse effects of drought on rice, supporting key physiological functions, including protein synthesis, enzyme activity, photosynthesis, water regulation, and stomatal control [24].

Although proline is known to play a significant role in plant growth and development under various environmental stresses, its specific impact on maize plants during drought stress remains underexplored. We hypothesized that proline supplementation could alleviate the detrimental effects of drought stress on maize by enhancing its resilience. The primary objective of this study was to investigate the role of proline in modulating key morpho-physiological and biochemical parameters in maize exposed to drought conditions. Specifically, we aimed to assess whether proline could improve growth, antioxidant enzymatic activity, water retention, nutrient balance, and photosynthetic efficiency, thereby promoting overall plant performance and stress tolerance. The results of this study provide valuable insights into the use of proline as a strategy for enhancing drought resistance in maize.

## 2. Materials and Methods

### 2.1. Experimental Setup

This study was conducted in a greenhouse in March 2023, using the *Zea mays* L. (B73 inbred line) cultivar. Four distinct treatment groups were established: (1) a control group treated only with water, (2) a group treated with proline supplementation (Proline), (3) a group subjected to drought stress alone (Drought), and (4) a group subjected to both drought stress and proline treatment (Drought + Proline). 

Surface-sterilized maize seeds were placed on moist filter paper and incubated in the dark at 25 °C for 48 h to initiate germination, as previously described [25]. Each germinated seed was then planted in a plastic pot containing 2 kg of a uniform soil mixture of peat, perlite, and sand in equal proportions. One seed was planted per pot, with each treatment replicated in triplicate. After one week of growth in pots, the Proline group was treated with 500 mL of a 30 mM proline solution. Drought stress was induced a week later by applying 500 mL of a 30% polyethylene glycol 6000 (PEG-6000) solution. Throughout the experiment, control plants were watered every other day, whereas the proline- and PEG-treated groups were supplied with fresh solutions daily, ensuring the previous day’s solution was drained before each new application. The treatment regimen continued for three weeks, adhering to the protocol outlined in [26]. 

This study measured a comprehensive set of phenotypic and biochemical parameters to evaluate the effects of the treatments on plant growth and resilience. Key variables measured included root and shoot lengths (averaging the longest and middle roots), fresh and dry weights, relative water content, nutrient composition, and antioxidant activity. At the end of the experiment, final assessments were carried out for root and shoot heights, as well as for fresh and dry biomass, to thoroughly evaluate the effects of the treatments. Dry weights were determined by drying the roots and shoots at 80 °C for 48 h [26].

### 2.2. Electrolyte Leakage Measurement

Electrolyte leakage (EL) in maize leaves under drought stress was assessed by collecting leaf samples one week after exposure to drought conditions. Approximately 200 mg of leaf tissue was cut into 5 mm pieces and placed in 10 mL of deionized water in closed test tubes. The samples were incubated at 32 °C for 2 h to facilitate the initial diffusion of electrolytes. Subsequently, the initial conductivity (EC1) was measured using a conductivity meter (CM-115, Kyoto Electronics, Kyoto, Japan). The samples were then autoclaved and cooled to room temperature, and their final conductivity (EC2) was measured. Finally, EL was calculated using the following formula:

$$EL=\frac{EC1}{EC2}\times 100$$

### 2.3. Measurement of Relative Water Content (RWC), Leaf Area, and Stem Diameter

The RWC of the leaves was calculated by subtracting the dry weight from the fresh weight of the leaves. Fresh weight was measured immediately after cutting the leaves from plants, while dry weight was measured after drying the leaves in an oven at 70 °C for 48 h. The leaf area for each treatment group was measured in triplicate by multiplying the leaf length, width, and a constant factor of 0.75, reflecting the average variation in maize leaf shape and curvature based on previous studies [27]. The total leaf area per plant (Yp, in cm^2^) was calculated by summing the areas of all individual leaf blades. A Vernier caliper was used to measure the diameter of the stem of each treatment group.

### 2.4. Chlorophyll Content

To assess the photosynthetic efficiency of maize plants under drought stress, their chlorophyll content was measured using a SPAD meter (SPAD-502 Plus; Konica Minolta Sensing, Seoul, Republic of Korea) after three weeks of stress exposure. Three leaves from each plant in every treatment group were selected for analysis. Chlorophyll measurements were taken at three different points on each leaf, namely the tip, the middle, and the base, to account for variability along the leaf.

### 2.5. Measurement of Hydrogen Peroxide and Lipid Peroxidation

The H_2_O_2_ concentration in maize plants was determined following a previously described method [28], with slight modifications. Briefly, 200 mg of fresh maize leaves was ground in liquid nitrogen, and H_2_O_2_ was extracted using 500 µL of 0.1% trichloroacetic acid (TCA). The extract was centrifuged at 10,000 rpm for 15 min. The resulting supernatant was mixed with 1 mL of 1 mM potassium iodide and 500 µL of 10 mM phosphate buffer, followed by centrifugation at 10,000 rpm for 15 min. The absorbance of the samples was measured at 390 nm using a spectrophotometer, and the H_2_O_2_ concentration was calculated using the extinction coefficient (ε = 0.28 mM/cm). 

Lipid peroxidation was evaluated by measuring malondialdehyde (MDA) levels using a commercially available kit from Sigma (Seoul, Republic of Korea). The detailed protocol for lipid peroxidation assessment is described in our previous study [29].

### 2.6. Measurement of Antioxidant Enzyme Activities

Catalase (CAT) and superoxide dismutase (SOD) activities were analyzed following the method described in a previous study [30]. For enzyme extraction, 1 g of fresh maize leaves was homogenized in 10 mL of 50 mM potassium phosphate (pH 7.0) containing 1 mM EDTA and 1% polyvinylpyrrolidone, followed by centrifugation at 12,000 rpm for 20 min. The supernatant was immediately used for enzyme activity assays. SOD activity was determined by measuring the extent of inhibition of the photochemical reduction of nitro blue tetrazolium at 560 nm. CAT activity was determined by monitoring the H_2_O_2_ decomposition rate at 240 nm. Peroxidase (POD) activity was measured using a previously established protocol [31]. Briefly, 0.2 g of fresh leaves was homogenized in 0.1 mM potassium phosphate buffer and then centrifuged at 12,000 rpm for 15 min. The resulting supernatant was used as the enzyme extract. The reaction mixture comprised 10 µL of enzyme extract, 1 mM potassium phosphate buffer, 50 µL of 50 µM pyrogallol, and 50 µL of 50 µM H_2_O_2_. After incubation at 25 °C for 5–10 min, the reaction was stopped by adding 5% H_2_SO_4_. The enzyme activity was measured at 420 nm using a spectrophotometer.

### 2.7. Measurement of Proline, Total Amino Acids, and Sugar Contents 

The proline content was quantified following the method described in a previous study [32]. The toluene-containing reaction mixture was analyzed spectrophotometrically at 520 nm, and the proline content was expressed as µg/g fresh weight (FW).

Total amino acids were extracted and measured using the protocol described in a previous study [33]. Briefly, fresh leaves were ground in liquid nitrogen, and approximately 0.2 mg of the sample was vacuum-hydrolyzed in 6 N HCl at 110 °C for 24 h and then dried at 80 °C for 24 h. Samples were reconstituted in 0.02 N HCl, filtered (0.45 µm filter paper), and analyzed using an amino acid analyzer (Hitachi, Tokyo, Japan, L-8900).

The total sugar content was determined as described previously [34], using high-performance liquid chromatography (HPLC) with an Aminex 87C column (300 × 7.8 mm; Bio-Rad, Hercules, CA, USA). Fresh leaf samples of maize (0.2 g) were homogenized in 80% ethanol and incubated at 80 °C for 20 min. Subsequently, the homogenate was centrifuged at 10,000 rpm for 15 min. The pellet was dissolved in 2 mL of water, filtered (0.2 µm filter paper), and analyzed using HPLC with water as the mobile phase at a flow rate of 0.6 mL/min.

### 2.8. Quantification of Nutrients (N, P, and K)

The nutrient content in the leaf samples was quantified using a methodology adapted from a previous study [35]. Leaves were randomly selected from three plants, dried, and finely crushed in liquid nitrogen for optimal homogenization. For digestion, the powdered samples were mixed with 3 mL of 62% HNO_3_ (*w*/*w*), 3 mL of 30% H_2_O_2_ (*w*/*w*), and 2 mL of 45% HF (*w*/*w*). The obtained mixtures were then subjected to microwave digestion to ensure complete dissolution. After digestion, the resulting solutions were diluted to a final volume of 100 mL with a 4% (*w*/*v*) boric acid solution. Finally, the nutrient content of the samples was measured using inductively coupled plasma (ICP) spectroscopy (Optima 7900DV; PerkinElmer, Waltham, MA, USA).

### 2.9. Statistical Analysis

The experiment followed a completely randomized design with three replications. Data were analyzed using one-way analysis of variance (ANOVA), followed by Bonferroni post hoc tests at *p* = 0.05. The results are presented as means and standard deviations, and they were visualized using GraphPad Prism (5.01; GraphPad Software, San Diego, CA, USA).

## 3. Results

### 3.1. Application of Proline Enhances Maize Growth Under Drought Stress

**Figure 1:**
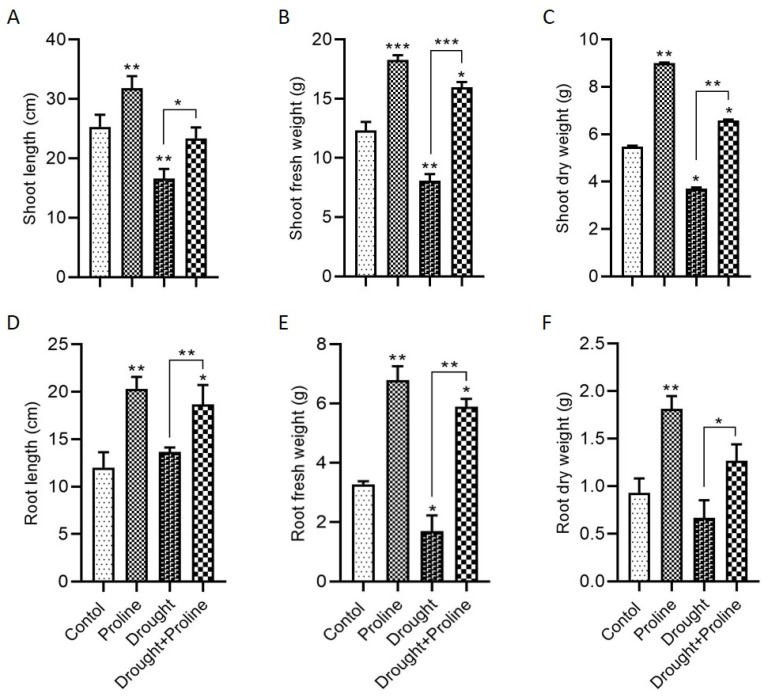
Proline application enhances maize growth parameters under drought stress. (**A**) Shoot length, (**B**) shoot fresh weight, (**C**) shoot dry weight, (**D**) root length, (**E**) root fresh weight, and (**F**) root dry weight. Significance levels are indicated as follows: * *p* < 0.05, ** *p* < 0.01, and *** *p* < 0.001. Bars represent standard error. Asterisks above the bars indicate comparisons with the control group, while asterisks above the lines denote comparisons between treatment groups.

Under standard, well-watered conditions, proline supplementation significantly improved several growth parameters. Specifically, maize plants treated with proline exhibited notable increases in both shoot and root lengths, as well as in the fresh and dry weights of the shoots and roots, compared to the untreated control plants (Figure 1). Under drought stress, the untreated plants displayed a considerable reduction in these growth metrics, emphasizing the detrimental impact of water deficiency on plant development. However, proline application under drought stress markedly alleviated these adverse effects. The proline-treated maize plants showed substantial improvements in growth parameters: shoot length increased by 40%, root length by 36%, shoot fresh weight by 97%, root fresh weight by 247%, shoot dry weight by 77%, and root dry weight by approximately 154% compared to the untreated drought-stressed plants.

### 3.2. Effects of Proline on Electrolyte Leakage, Water Retention, Leaf Area, Stem Diameter, and Chlorophyll Stability

**Figure 2:**
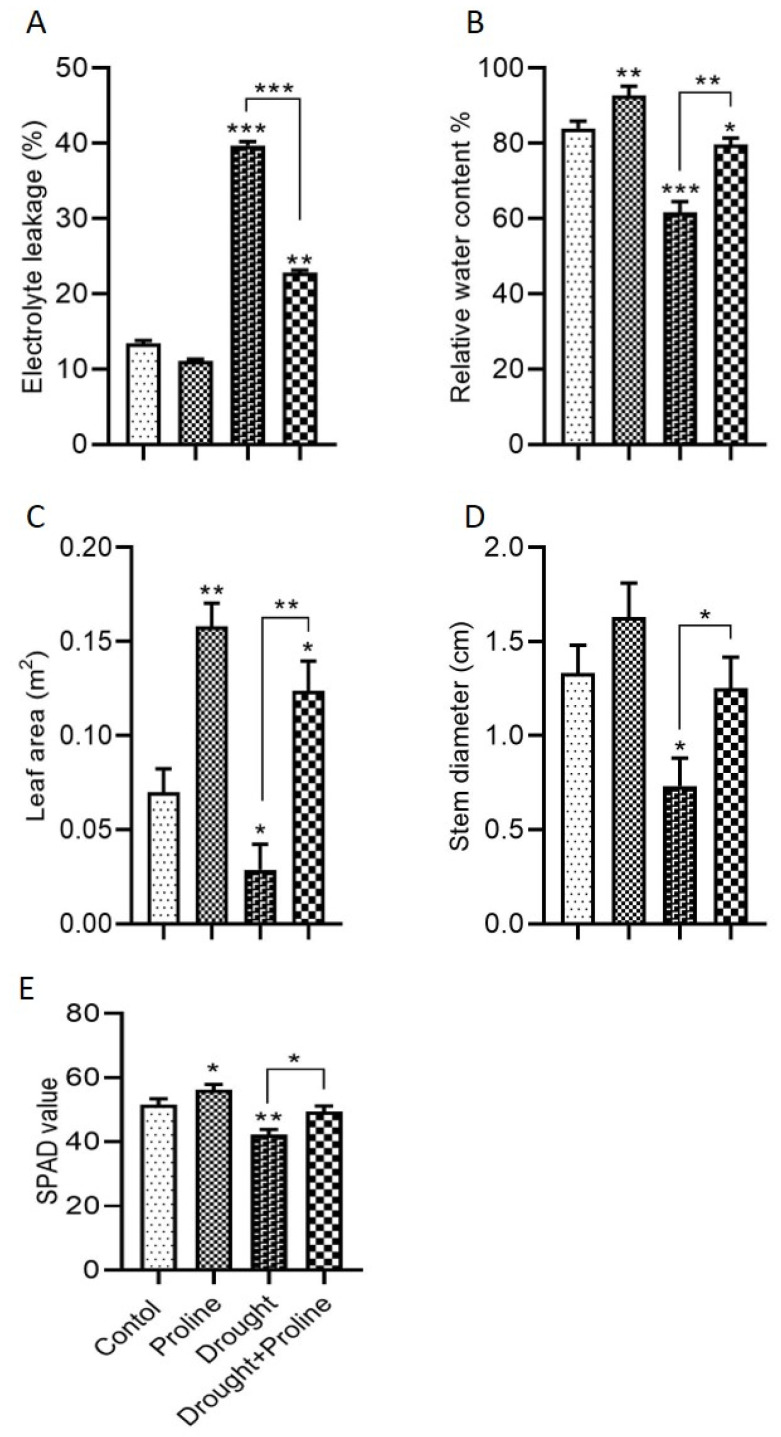
Proline application enhances physiological parameters in maize under drought stress. (**A**) Electrolyte leakage, (**B**) relative water content, (**C**) leaf area, (**D**) stem diameter, and (**E**) chlorophyll content (SPAD value). Significance levels are denoted as follows: * *p* < 0.05, ** *p* < 0.01, and *** *p* < 0.001. Bars represent standard error. Asterisks above the bars indicate comparisons with the control group, while asterisks above the lines denote comparisons between treatment groups.

Drought stress increased electrolyte leakage by 195% under normal conditions compared to the control plants (Figure 2A). However, proline supplementation significantly reduced electrolyte leakage by 42% under drought conditions compared to the untreated drought-stressed plants. Electrolyte leakage typically rises under stress due to cell membrane damage from oxidative stress, lipid peroxidation, and dehydration. Our results indicate that proline supplementation mitigates these effects by reducing oxidative stress, decreasing lipid peroxidation, and enhancing water retention in cells, thereby improving overall membrane stability. Drought stress also reduced the RWC by 26% compared to the control plants, while proline supplementation under drought conditions restored the RWC by 29% relative to the untreated drought-stressed plants (Figure 2B). A similar trend was observed in leaf area; drought stress reduced leaf area compared to the controls, whereas proline significantly increased leaf area under drought stress relative to the untreated drought-stressed plants (Figure 2C). Proline also improved stem diameter under drought conditions, further indicating its protective role (Figure 2D). Moreover, proline supplementation enhanced photosynthetic efficiency by preserving the chlorophyll content during drought stress. The chlorophyll content increased by 8% and 17% with proline supplementation under normal and drought conditions, respectively, compared to the control and untreated drought-stressed plants (Figure 2E).

### 3.3. Proline Mitigates Drought-Induced Oxidative Stress by Regulating the Antioxidant System

**Figure 3:**
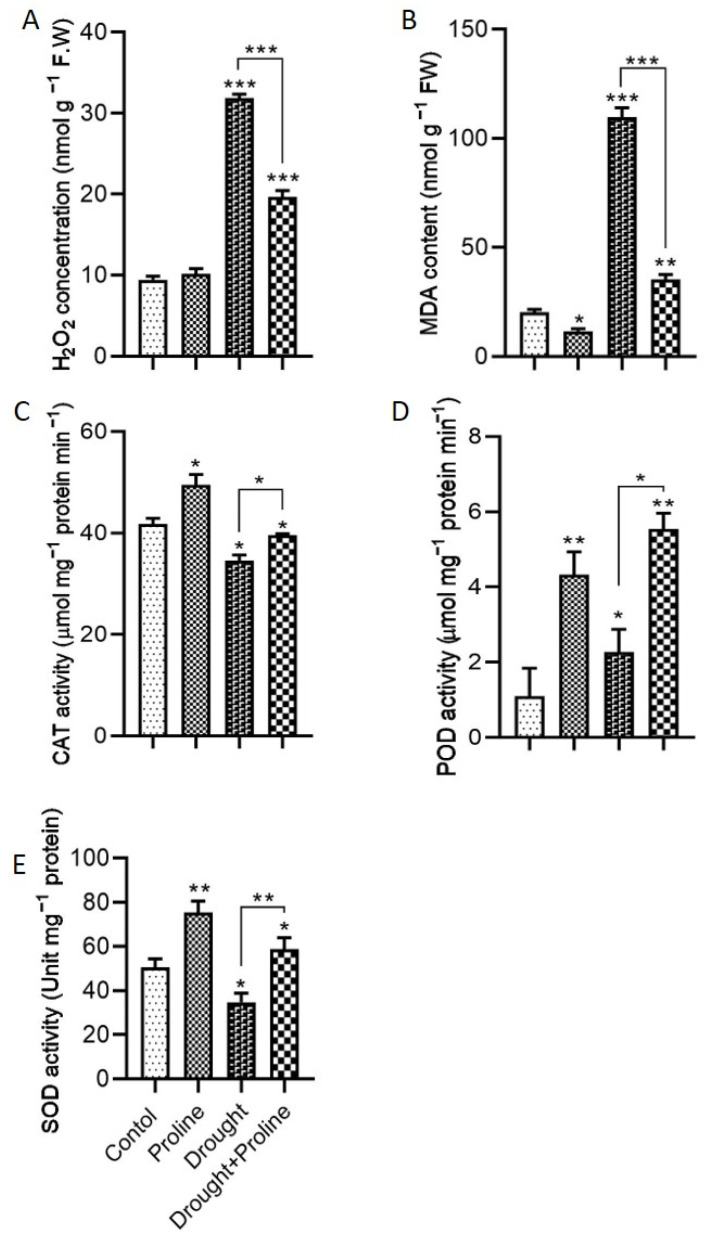
Effects of proline application on oxidative stress markers and antioxidant enzyme activities in maize under drought stress. (**A**) H_2_O_2_ concentration, (**B**) malondialdehyde (MDA) concentration, (**C**) catalase (CAT) activity, (**D**) peroxidase (POD) activity, and (**E**) superoxide dismutase (SOD) activity. Significance levels are denoted as follows: * *p* < 0.05, ** *p* < 0.01, and *** *p* < 0.001. Bars represent standard error. Asterisks above the bars indicate comparisons with the control group, while asterisks above the lines denote comparisons between treatment groups.

To elucidate the mechanism behind proline’s mitigation of drought-induced oxidative stress, we assessed H_2_O_2_ accumulation, antioxidant enzyme activity, and lipid peroxidation levels (Figure 3). Drought stress, which is known to trigger oxidative stress by producing ROS like H_2_O_2_ and O_2_, significantly elevated H_2_O_2_ levels in the plants. Specifically, the drought-stressed plants displayed markedly higher H_2_O_2_ accumulation compared to the control plants, indicating increased ROS production. Notably, proline supplementation under drought stress conditions effectively reduced H_2_O_2_ levels by approximately 38% compared to the untreated drought-stressed plants (Figure 3A), suggesting that proline alleviates oxidative stress by limiting ROS generation during drought. Furthermore, this study measured the MDA concentration as an indicator of lipid peroxidation, a process that reflects membrane damage under stress. Proline-treated plants subjected to drought stress exhibited a 67% reduction in MDA levels compared to the untreated drought-stressed plants (Figure 3B).

Additionally, we investigated the activity of the key antioxidant enzymes CAT, POD, and SOD to understand proline’s impact on the plant’s antioxidant defense system. In the absence of drought stress, the proline-treated plants showed a significant increase in CAT, POD, and SOD activities relative to the control plants, indicating enhanced basal antioxidant activity. Under drought stress, the activities of CAT, POD, and SOD in the proline-treated plants increased by 14%, 69%, and 144%, respectively, compared to the drought-stressed plants without proline supplementation (Figure 3C–E). These findings suggest that proline enhances the plant’s enzymatic antioxidant defenses, mitigating oxidative damage caused by ROS accumulation under drought conditions. Overall, our results indicate that proline supplementation reduces oxidative stress in maize plants subjected to drought by modulating ROS accumulation, minimizing lipid peroxidation, and enhancing antioxidant enzyme activity.

### 3.4. Proline Enhances Total Protein and Nutrient Contents Under Drought Stress

**Figure 4:**
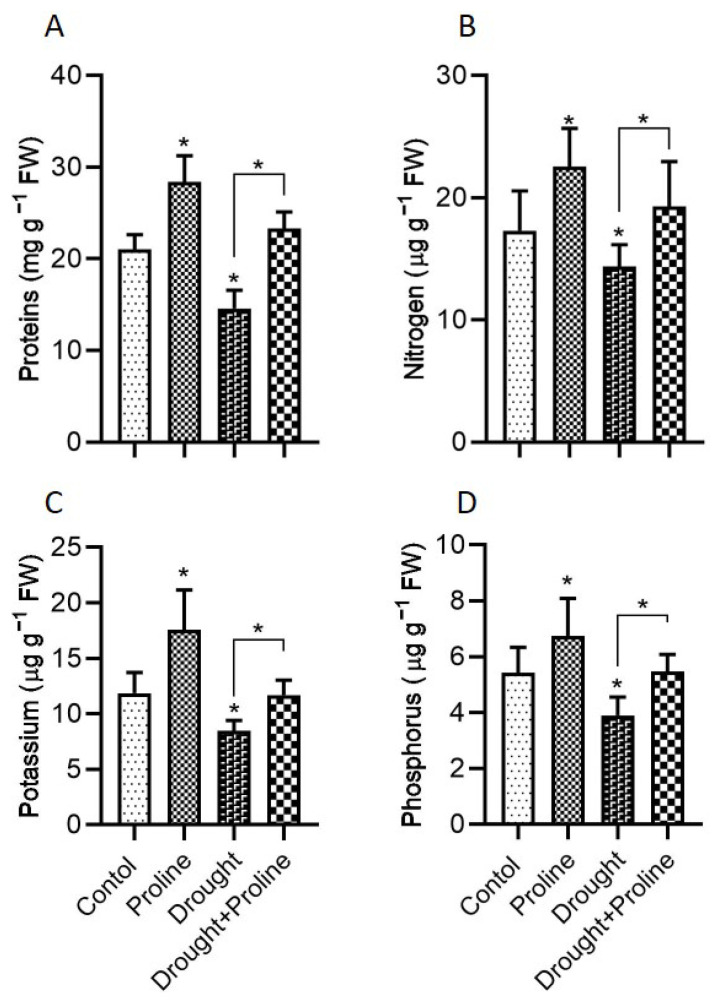
Proline application enhances protein and nutrient concentrations in maize plants under drought stress. (**A**) Protein, (**B**) nitrogen, (**C**) potassium, and (**D**) phosphorus. Drought stress significantly reduced the levels of protein, nitrogen, potassium, and phosphorus. However, proline application under drought stress increased these levels, indicating a mitigative effect on nutrient depletion due to drought. Data are presented as mean + standard error. Significance levels are denoted as * *p* < 0.05. Bars represent standard error. Asterisks above the bars indicate comparisons with the control group, while asterisks above the lines denote comparisons between treatment groups.

Protein levels play a crucial role in plant responses to drought stress, acting as a reservoir of functional and structural molecules essential for resilience under stress conditions. To assess the impact of drought and proline supplementation on protein accumulation, total protein levels were quantified across all treatment groups (Figure 4A). Proline supplementation under normal conditions significantly increased total protein concentrations compared to the control plants, suggesting that proline positively influences protein synthesis in maize. Our findings further demonstrated that drought stress led to a notable reduction in total protein concentrations compared to the control plants, indicating drought’s adverse effect on protein stability and synthesis. However, proline treatment mitigated this impact, with the drought-stressed plants supplemented with proline exhibiting a 60% increase in protein concentration relative to the drought-stressed plants without proline treatment.

Additionally, we analyzed the accumulation of key nutrients—nitrogen, potassium, and phosphorus—as they play critical roles in metabolic and osmotic balance under stress. Under normal conditions, the proline-treated plants exhibited significantly higher N, K, and P levels than the control plants, indicating proline’s role in nutrient uptake or retention (Figure 4B–D). Under drought stress, nutrient levels significantly declined, reflecting the limited nutrient availability or uptake often associated with water deficit. However, the proline-supplemented plants under drought stress maintained their nutrient levels, showing a 30%, 40%, and 28% increase in N, K, and P concentrations, respectively, compared to the untreated drought-stressed plants.

### 3.5. Proline and Sugar Accumulation During Drought Stress

**Figure 5:**
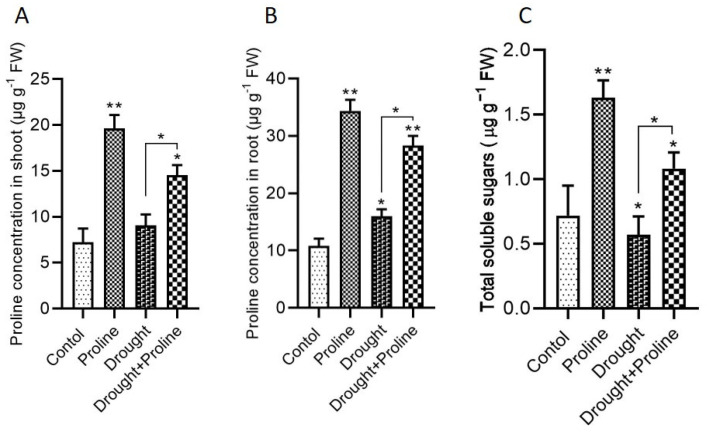
Exogenous application of proline enhances endogenous proline and sugar levels in maize plants under drought stress. (**A**) Proline concentration in shoots, (**B**) proline concentration in roots, and (**C**) total soluble sugars. Data are presented as mean + standard error. Significance levels are denoted as follows: * *p* < 0.05 and ** *p* < 0.01. Bars represent standard error. Asterisks above the bars indicate comparisons with the control group, while asterisks above the lines denote comparisons between treatment groups.

Our study demonstrated that, under both normal and drought-stressed conditions, the proline-treated plants exhibited a significant increase in proline concentration in both the shoots and roots compared to the untreated plants (Figure 5A,B). In the shoots, proline accumulation increased by 172% in the proline-treated plants under normal conditions compared to the control, while the drought-stressed plants supplemented with proline showed a 60% increase compared to the untreated drought-stressed plants. This elevated proline accumulation likely contributed to osmotic adjustment, helped to maintain cell turgor, and mitigated the impact of water deficit stress. In addition to enhancing proline levels, proline supplementation also influenced the accumulation of soluble sugars. Under both normal and drought-stressed conditions, the proline-treated plants showed a significant increase in sugar concentration compared to the control plants (Figure 5C). Specifically, proline application resulted in a 90% increase in sugar concentration in the drought-stressed plants compared to the untreated drought-stressed controls.

## 4. Discussion

**Figure 6:**
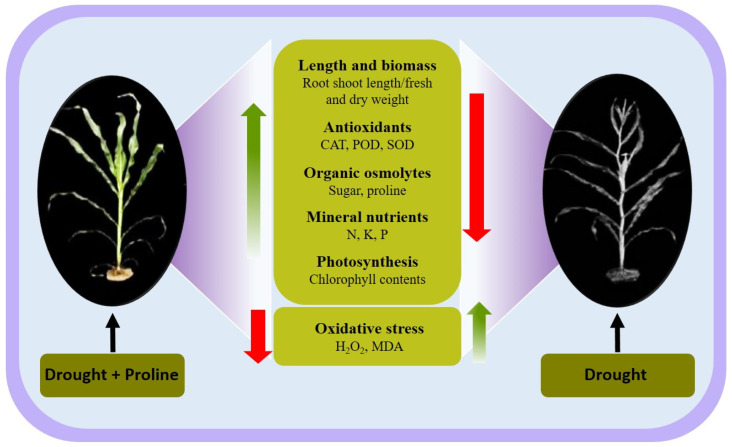
This figure illustrates maize growth responses under drought stress with and without proline supplementation. The left panel shows a maize plant treated with both drought stress and exogenous proline, displaying improved growth parameters. In contrast, the right panel depicts a maize plant that was subjected to drought stress alone, exhibiting reduced growth and chlorophyll content. The central part shows the physiological and biochemical changes observed under these conditions. Proline treatment enhances growth metrics and antioxidant activities, including catalase (CAT), peroxidase (POD), and superoxide dismutase (SOD), as well as the accumulation of organic osmolytes, such as sugars and proline. Additionally, it increases the concentrations of essential mineral nutrients, including N, P, and K. These changes collectively support photosynthesis and reduce oxidative stress indicators, such as H_2_O_2_ and malondialdehyde (MDA). The green arrows indicate increases, while the red arrows indicate decreases in response to proline supplementation under drought stress.

Drought stress significantly limits maize productivity by impairing various physiological, biochemical, and molecular processes. Our study demonstrates that proline supplementation enhances maize resilience to drought by improving growth, water retention, membrane stability, and nutrient accumulation while reducing oxidative damage (Figure 6 and Figure S1). These findings highlight proline’s multifaceted role in mitigating drought stress and offer valuable insights for developing strategies to improve crop resilience under challenging climate conditions.

Plants employ several adaptive mechanisms to cope with environmental stresses, particularly abiotic stresses like drought. The application of osmoprotectants, such as amino acids, proline, betaines, and trehalose, strengthens plant resilience by reducing stress-related damage [36]. In this study, we investigated the effect of exogenous proline on maize plants under drought conditions, demonstrating its role as an efficient osmolyte. Proline supplementation significantly improved plant growth parameters, including shoot and root lengths, fresh and dry weights, leaf area, and stem diameter, under drought stress. Importantly, exogenous proline application increased endogenous proline accumulation in both the roots and shoots, further supporting growth under drought conditions. In contrast, drought stress alone severely impaired these growth parameters. These results align with previous studies showing that exogenous proline application in maize promotes endogenous proline accumulation and enhances growth, mitigating the detrimental effects of drought stress [8]. This is consistent with findings in rice and *Allenrolfea occidentalis*, where proline supplementation enhanced growth under both drought and salt stress [37,38]. Exogenously applied proline boosts endogenous proline levels, stabilizing enzymes, proteins, and membrane components, while potentially providing energy for growth and stress tolerance during drought [8]. Similar to drought stress, exogenous proline supplementation has been shown to enhance plant growth and biomass under salt stress in various species, such as *Medicago sativa*, *Helianthus annuns*, *Cucumis sativus*, *Triticum durum*, *Zea mays*, and *Oryza sativa* [39,40,41,42,43,44]. However, the impact of exogenous proline can vary depending on the plant species, developmental stage, application method, and concentration. In our study, applying 30 mM proline at the seedling stage significantly improved maize growth parameters, mirroring findings in rice, where 30 mM proline enhanced growth at the seedling stage. Comparable results have been reported with 30 mM proline in mung bean cell cultures, while 10 mM was effective in tobacco suspension cells [45,46]. These findings highlight the potential of proline as a versatile osmoprotectant that enhances plant growth and resilience under drought conditions, especially when applied at optimal concentrations and developmental stages. Further research could explore the underlying mechanisms of proline-induced stress tolerance, offering valuable insights for developing sustainable crop management practices in stress-prone environments.

In maize, drought stress markedly affects photosynthesis by reducing leaf area and chlorophyll levels, both of which are crucial for maintaining high photosynthetic efficiency. In our study, limited water availability led to a decline in chlorophyll levels, primarily due to increased oxidative stress, resulting in elevated H_2_O_2_ and MDA levels, as well as reduced activity of antioxidant enzymes, including CAT, POD, and SOD (Figure 3). However, exogenous application of proline effectively alleviated oxidative stress, enhancing antioxidant enzyme activities and thereby mitigating drought-induced damage. Additionally, proline anabolism can enable plants to maintain osmotic balance, facilitating the recovery of water content, especially during osmotic stress [47]. Similarly, Qasim Ali et al. demonstrated that proline application supports photosynthesis in maize under drought conditions by mitigating oxidative stress [8]. Our findings further suggest that proline application reduces H_2_O_2_ production and MDA accumulation (Figure 3A,B), which in turn enhances stomatal conductance and CO_2_ assimilation, thereby promoting photosynthesis. These observations are in line with previous studies showing that foliar application of proline in drought-stressed maize improves stomatal conductance and CO_2_ uptake, supporting photosynthetic processes [48].

Additionally, our study indicates that exogenous proline increases cellular proline levels, which stimulates the activities of antioxidant enzymes, such as CAT, POD, and SOD, effectively reducing oxidative stress. Several studies have suggested that proline functions as an electron acceptor, boosting antioxidant enzyme activity and minimizing oxidative damage [49,50,51]. For example, in sugar beet, exogenous proline has been shown to increase the levels of endogenous proline and phenolic compounds, as well as antioxidant enzyme activities, helping to alleviate oxidative stress symptoms [52]. Furthermore, numerous studies have indicated that proline application can boost stress tolerance by improving nutrient acquisition, water uptake, photosynthesis, gas exchange, and antioxidant responses [47,53,54,55]. In our study, proline application under drought conditions mitigated the buildup of H_2_O_2_ and MDA by enhancing antioxidant enzyme activities. Similar results were observed in recent studies, where seed priming with proline enhanced ascorbate peroxidase (APX), CAT, and SOD activities in maize cultivars FS-67 and CML-539 [56]. Notably, CAT triggers various responses that help plants adapt to stress, while SOD acts as a frontline defense by quickly converting ROS into safer molecules, such as oxygen and water [57]. A recent study also indicated that proline acts as an ROS scavenger, protecting chloroplasts and nuclei from oxidative damage [58]. These results highlight the potential of proline as a valuable osmoprotectant for alleviating drought-induced oxidative stress, enhancing photosynthetic efficiency, and improving overall plant resilience under water-deficit conditions.

Under drought stress, plants employ various adaptive mechanisms to maintain cell turgor, one of which is osmotic regulation. This process involves the accumulation of osmoprotectants, such as proline, soluble sugars, and amino acids, to help retain water and preserve cellular function. Studies have shown that osmolytes play a vital role in plant defense by enabling cells to retain water via osmotic adjustment, thereby preserving turgor pressure [59,60]. In our study, maize seedlings treated with exogenous proline under drought conditions exhibited significantly higher levels of total protein, soluble sugars, and essential nutrients (N, P, and K), along with increased intercellular proline, compared to the untreated drought-stressed plants (Figure 4 and Figure 5). Although some studies suggest that drought stress alone can elevate the levels of proteins, sugars, and amino acids compared to those observed in irrigated plants [55], our findings revealed a reduction in these metabolites in plants subjected solely to drought stress compared to the control plants. This discrepancy may be attributed to the higher severity or prolonged duration of stress in our study, potentially overwhelming the plants’ adaptive mechanisms. Differences in plant responses to drought stress across studies are likely influenced by factors such as plant species, developmental stage, environmental conditions, and experimental designs, all of which shape metabolic adaptations to stress. In the present study, proline-primed maize seeds accumulated elevated levels of endogenous proline and sugars during drought conditions [16], consistent with other studies indicating that exogenous proline application increases endogenous proline levels in water-stressed plant tissues, enhancing osmotic adjustment [8,36,61,62].

Our results also align with recent studies demonstrating that exogenous proline promotes the uptake of potassium, calcium, phosphorus, and nitrogen under drought stress [23], highlighting the importance of nutrient balance for drought tolerance. Potassium is essential for osmoregulation, enzyme activation, and stomatal regulation, while nitrogen contributes to cellular structures, and phosphorus plays a central role in energy transfer, respiration, and photosynthesis [63,64]. Notably, in our study, drought stress without proline supplementation significantly reduced the levels of these key nutrients (N, P, and K). Pessarakli [65] attributed such reductions to lower respiration rates and stomatal conductance during water stress. Given that nutrient uptake in plants depends on water movement from the roots to the shoots [66], our findings suggest that exogenous proline application mitigated drought stress in maize by promoting nutrient absorption and accumulation. This suggests that proline application may serve as an effective strategy for improving drought tolerance in maize by improving the levels of essential nutrients under water-limited conditions.

## 5. Conclusions

This study demonstrates the positive impact of exogenous proline application in enhancing maize resilience under drought stress. Proline treatment improved growth metrics, including shoot and root lengths, fresh and dry weights, and chlorophyll contents, while reducing electrolyte leakage and oxidative stress indicators (H_2_O_2_ and MDA). This was achieved through the activation of antioxidant enzymes (CAT, POD, and SOD). Additionally, proline enhanced protein concentrations and macronutrient (N, P, and K) levels, promoting drought tolerance by improving metabolic processes and osmotic balance. The accumulation of endogenous proline and sugars further contributed to osmoprotection and alleviated drought-induced damage. Despite these promising results, the present study has limitations in fully understanding the underlying molecular mechanisms. Future research should focus on exploring the molecular pathways through which proline enhances antioxidant activity and nutrient accumulation in maize and other crops.

## Data Availability

The data supporting the findings of this study are available in the Supplementary Materials of this article.

## Supplementary Materials

The following supporting information can be downloaded at: https://www.mdpi.com/article/10.3390/biology14010041/s1, Figure S1: Proline Enhances Growth and Stress Tolerance in Maize under Drought Conditions.
