# Supplementary material for: Proline Promotes Drought Tolerance in Maize
Source: Biology (Basel). 2025 Jan 7;14(1):41. doi: 10.3390/biology14010041 (PMC11762158; doi:10.3390/biology14010041)
Supplement: Supplementary file 1 [file biology-14-00041-s001.zip › biology-3348966-supplementary.pdf]

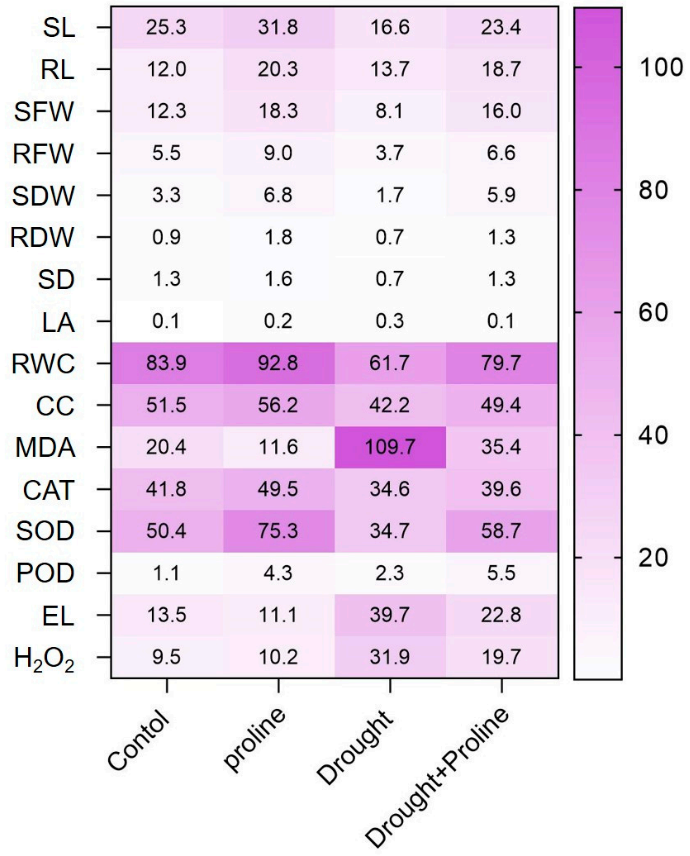

Figure S1. Figure illustrates the impact of proline on growth and stress-related parameters under normal and drought conditions. Proline supplementation under drought stress mitigated growth reductions in shoot and root length, fresh and dry weights, highlighting its growth-promoting effect. Relative water content (RWC) and chlorophyll content (CC) were also improved with proline, indicating better water retention and photosynthetic activity. Oxidative stress markers, MDA and H_2O_2, were elevated under drought but reduced with proline application, which also enhanced antioxidant enzyme activities (CAT, SOD, and POD). These findings suggest that proline strengthens the plant's antioxidant defense, reducing oxidative damage. Additionally, reduced electrolyte leakage (EL) under drought + proline treatment reflects improved membrane stability. Overall, proline plays a protective role in enhancing growth and resilience under drought stress.".
